# Functional MRI study of neurovascular coupling in patients with non-lesional epilepsy

**DOI:** 10.3389/fnhum.2024.1517565

**Published:** 2025-01-22

**Authors:** Zhisen Li, Xiaoxia Hou, Yanli Lu, Huimin Zhao, Meixia Wang, Qian Gui, Guanhui Wu, Qinrong Xu, Xiaofeng Dong, Qingzhang Cheng, Xiaowen Xu, Hongxuan Feng

**Affiliations:** ^1^Department of Radiology, The Affiliated Suzhou Hospital of Nanjing Medical University, Suzhou Municipal Hospital, Suzhou, China; ^2^Department of Neurology, The Affiliated Suzhou Hospital of Nanjing Medical University, Suzhou Municipal Hospital, Suzhou, China; ^3^Department of Emergency, The Affiliated Suzhou Hospital of Nanjing Medical University, Suzhou Municipal Hospital, Suzhou, China

**Keywords:** epilepsy, neurovascular coupling, resting-state functional magnetic resonance imaging, arterial spin labeling, amplitude of low-frequency fluctuation

## Abstract

**Objective:**

The diagnosis of patients with non-lesional epilepsy (NLE) is relatively challenging because of the absence of a clear focus on imaging, and the underlying pathological mechanism remains unclear. The neuronal activity and functional connectivity of NLE patients are significantly abnormal, and the neuronal activity of epilepsy patients is closely related to cerebral blood flow (CBF). Neurovascular coupling (NVC) offers insights into the relationship between neuronal activity and CBF. Hence, we intend to explore the alterations of NVC in NLE patients and their influences on cognitive function.

**Methods:**

Clinical data of 24 patients with NLE (15 female; age range 19–40 years; median age 30.5 years) and 39 healthy controls (27 female; age range 19–40 years; median age 30 years) were collected, and resting-state functional magnetic resonance imaging (rs-fMRI) and 3D arterial spin labeling (ASL) were performed. The imaging indexes of amplitude of low-frequency fluctuation (ALFF) and CBF were calculated, respectively, by post-processing analysis. The differences in CBF, ALFF and CBF/ALFF ratio between the two groups were analyzed, along with correlation with clinical data of NLE patients.

**Results:**

Compared with the healthy controls, the CBF of the right parahippocampal gyrus was significantly decreased, and the CBF/ALFF ratio of the right inferior parietal, but supramarginal and angular gyri was significantly increased in NLE patients (*p* < 0.001). Moreover, the CBF/ALFF ratio was positively correlated with epilepsy depression score (*r* = 0.546, *p* = 0.006).

**Conclusion:**

NLE patients showed abnormal local NVC, which was associated with the severity of depression. The combined application of rs-fMRI and ASL can comprehensively evaluate the neuronal activity and cerebral blood perfusion in patients with NLE. The abnormal NVC is of great significance for us to explore the central mechanism of the occurrence and development of NLE.

## 1 Introduction

Epilepsy is a disease of the central nervous system due to the abnormal discharges of neurons. The latest research shows that the number of patients with epilepsy worldwide is as high as 70 million, and the clinical manifestations of patients with epilepsy are complex and diverse, often accompanied by neuropsychiatric symptoms such as cognitive impairment, mood abnormalities, depression and anxiety (Beghi, 2020; Mula et al., 2022), which seriously affects the health and quality of life of patients. The diagnosis of epilepsy not only depends on the clinical symptoms and electrophysiological examination, magnetic resonance imaging (MRI) also provides important information for the diagnosis and treatment of epilepsy. Epilepsy can be due to lesional epilepsy (Mofatteh et al., 2024) and non-lesional epilepsy (NLE) (Zhang et al., 2011). The NLE is defined as no clear lesions in routine imaging examinations (such as CT, MRI, etc.). The clinical diagnosis of NLE is relatively difficult, and the location of the epileptic focus and the selection of treatment plan are still a major problem faced by clinicians. Antiepileptic drugs are commonly used in the treatment of NLE patients (Sheng et al., 2018). Despite the better effect of drugs on the control of seizures, 1/3 patients with epilepsy still develop drug resistance (Sheng et al., 2018; Foit et al., 2020). The lack of definite epileptogenic foci makes the surgical treatment of NLE patients very difficult. Therefore, exploring the underlying pathological mechanisms of NLE occurrence and development is of great significance for clinical diagnosis and treatment.

The rapid development of resting-state functional magnetic resonance imaging (rs-fMRI) provides a new method for the study of the pathological mechanism of epilepsy. The author’s previous studies on the brain structure and function of patients with NLE found that patients with NLE had obvious abnormal neuronal activity and functional connectivity (Li et al., 2022). Meanwhile, neuronal activity in epilepsy patients is closely related to cerebral blood flow (CBF) (Lanting et al., 2009). Amplitude of low-frequency fluctuation (ALFF) is one of the common research methods for rs-fMRI, which obtaining the oscillation amplitude of the whole brain blood oxygenation level-dependent (BOLD) signal within a frequency band (0.01–0.08 Hz) and the spontaneous activity intensity of each voxel in the resting state is evaluated. Arterial spin labeling (ASL) can noninvasively and quantitatively evaluate the CBF of the whole brain and regions. At present, most of the studies on epilepsy use a single imaging method, which cannot fully and accurately reflect the changes of local CBF and neuronal activity caused by seizures.

Neurovascular unit (NVU) is the basic unit of brain structure and function, mainly composed of neurons, glial cells, vascular endothelial cells and extracellular matrix (Hu et al., 2019). The supply–demand balance and tight coupling between neuronal activity and cerebral blood flow play an important role in NVU. The combination of rs-fMRI and ASL can noninvasively and comprehensively evaluate the changes in neurovascular coupling (NVC) in patients with NLE, and re-understand the relationship between neuronal activity and regional cerebral blood flow. At present, neurovascular coupling has been studied in the aspects of mental diseases (Zhu et al., 2015), diabetes (Zhang et al., 2021) and neurodegenerative diseases (Zheng et al., 2019), and it has been confirmed that neurovascular decoupling may be the pathophysiological mechanism of these diseases. Therefore, we plan to jointly apply rs-fMRI and ASL to study the NVC changes and its effects on cognitive function in NLE patients, in order to further understand the pathological mechanism of NLE from a new perspective.

## 2 Materials and methods

### 2.1 Subjects

Twenty-four NLE patients diagnosed by the Department of Neurology, Suzhou Hospital Affiliated to Nanjing Medical University from June 2021 to March 2023 were included in this study (one patient was excluded due to excessive head movement during MRI examination). Eligibility criteria: (1) patients met the 2017 International League Against Epilepsy (ILAE) diagnostic criteria for epilepsy (Fisher et al., 2017); (2) right-handed; (3) no disease affecting brain structure and function; (4) no somatic or mental diseases; (5) no contraindications to MRI examination, and able to complete all examinations. During the same period, 39 healthy volunteers matched with the NLE group in gender, age and education level were recruited as the healthy control group. The healthy control group had no mental and neurological history, and no clear intracranial lesions in MRI examination. All enrolled subjects were informed of the purpose and content of the study, and signed the informed consent voluntarily. This study was approved by the Ethics Committee of Suzhou Hospital Affiliated to Nanjing Medical University.

General clinical data such as age, gender and education level were collected from all NLE patients and the healthy controls. A series of neuropsychological scales, including Hamilton Anxiety Scale (HAMA) and Hamilton Depression Scale (HAMD) were used to evaluate the subjects’ mental and emotional state within one week after MRI scanning by an experienced neurologist.

### 2.2 Imaging equipment and scanning sequence

Patients with NLE and healthy controls underwent MRI examination. Routine MRI, resting-state BOLD and ASL sequence were performed by Siemens Skyra 3.0 T MR scanner and 8-channel head coil. All subjects were awake, quiet and closed their eyes during the examination. The conventional MRI sequences included: axial T1WI, T2WI, FLAIR and DWI, and sagittal T2WI. The parameters of BOLD-fMRI: TR: 2720 ms, TE: 30.0 ms, slice thickness: 3.0 mm, slice spacing: 0 mm, FOV: 192 mm × 192 mm, voxel size: 2.0*2.0*3.0 mm. The parameters of ASL sequence: TR: 5000 ms, TE: 36.34 ms, TI: 1990 ms, Bolus Duration: 700 ms, slice thickness: 3.0 mm, slice spacing: 0 mm, Flip angle: 90°, FOV: 192 mm × 192 mm, voxel size: 3.0*3.0*3.0 mm. The parameters of three-dimensional (3D) T1-weighted sequence: TR: 2300 ms, TE: 2.98 ms, TI: 900 ms, slice thickness: 1.1 mm, slice spacing: 0 mm, Flip angle: 9°, FOV: 256 mm × 256 mm, voxel size: 1.0*1.0*1.0 mm.

### 2.3 Preprocessing of BOLD and calculation of ALFF

SPM12 toolkit was used for post-processing analysis on the MATLAB 22a platform. The specific steps include: (1) format conversion; (2) removal of the first 5 time points; (3) time-layer correction; (4) head movement correction (deleting the subjects’ images with head movement more than 3 mm or 3°); (5) functional image registration; (6) spatial standardization (resampling voxel was 3 mm × 3 mm × 3 mm); (7) smoothing (Gaussian kernel was 4 mm half width and height); (8) de-linear drift; (9) head movement, cerebrospinal fluid signal, and white matter signal underwent linear regression. The time series of each voxel in the pre-processing image was converted into frequency range by Fourier transform to obtain power spectrum, and then summed the energy square root of each frequency point in the 0.01 ~ 0.08 Hz band to obtain ALFF value, and finally the standardized ALFF value was obtained by dividing the whole brain average.

### 2.4 Preprocessing of ASL and calculation of CBF

Based on the MATLAB 22a platform, SPM12 (http://www.fil.ion.ucl.ac.uk/spm) and ASLtbx toolkits were used for data processing analysis. The specific steps are as follows: (1) image reduction, setting the origin of each image as the center; (2) head movement correction, correcting the ASL image to the reference image, which was set as the first image of each subject; (3) registration, registering the ASL image after head movement correction with the structural image (T1) of each subject; (4) removal of extracranial voxel, generating mask file, generating Brain mask based on the average image of ASL image; (5) filtering and regression of noise covariates to remove low-frequency and high-frequency noise; (6) smoothing (smoothing kernel size 6 mm); (7) calculating CBF and normalizing it to the mean of the whole brain; (8) standardization and result statistics.

### 2.5 Analysis of NVC in the whole brain

To facilitate the calculation of coupling indexes, firstly, the CBF map was re-cut, and the size of the re-cut voxels was 3 mm × 3 mm × 3 mm, which was consistent with the ALFF map. Then, the CBF map and the ALFF map were normalized by z score (subtracting the mean value of the whole brain and dividing by the standard deviation) in the gray matter range. The cross-voxel Pearson correlation was calculated on the normalized CBF map and the ALFF map to obtain the across-voxel coupling value of each subject, and then the coupling value was transformed by Fisher’s z transform, and the two-sample T test was performed on the NLE group and the HC group after the transformation.

### 2.6 Analysis of NVC in the region

The CBF map and the ALFF map before normalization were used to obtain the CBF/ALFF Ratio map of each subject, and then the Ratio map was normalized by z score. The two-sample T test was performed on the normalized Ratio map of the NLE group and the HC group, and the results were corrected by FWE (Family-Wise Error) at the cluster level (voxel *p* < 0.001, cluster *p* < 0.05).

### 2.7 Statistical analysis

SPSS 22.0 statistical software was used for analysis. The independent sample *t* test was used to compare the continuous data (age, years of education, HAMA score, HAMD score) conforming to the normal distribution. The χ^2^ test was used compare the counting data expressed as frequency or percentage (gender). The CBF value, ALFF value and CBF/ALFF ratio between the NLE group and HC group were tested by independent sample *t* test. Gender, age and years of education were taken as covariables, and FWE correction was performed on the results at the cluster level (voxel *p* < 0.001, cluster *p* < 0.05). Pearson correlation analysis was performed between the indicators that had differences between the two groups and the HAMA and HAMD scores of NLE patients.

## 3 Results

### 3.1 Subjects’ clinical data and neuropsychological scores

Twenty-four patients with NLE and 39 healthy controls were included in the study. In our study, one enrolled subject was excluded due to excessive head movement during the examination. The main clinical data of NLE group and HC group are shown in Table 1. There were no significant differences in age, sex and years of education between the two groups (*p* > 0.05). HAMD scores in NLE group were higher than those in HC group, and the difference was statistically significant (*p* < 0.001).

**Table 1:** Comparison of baseline data.

Characteristics	NLE (*n* = 24)	HC (*n* = 39)	*t*/*χ*^2^ value	*p*-value
Age (years, age range)	31.21 ± 6.00 (19–40)	30.13 ± 5.28 (19–40)	0.749	0.457
Gender (male/female)	9/15	12/27	0.076	0.595
Education (years)	14.67 ± 2.55	13.95 ± 2.27	1.163	0.249
HAMA score	6.29 ± 2.27	4.59 ± 2.27	2.890	0.005*
HAMD score	14.75 ± 4.21	11.36 ± 3.12	3.656	0.001*

*p < 0.05; NLE, non-lesional epilepsy; HC, healthy controls; HAMA, Hamilton Anxiety Scale; HAMD, Hamilton Depression Scale.

### 3.2 Comparison of CBF, ALFF, and CBF/ALFF ratio between groups

**Figure 1:**
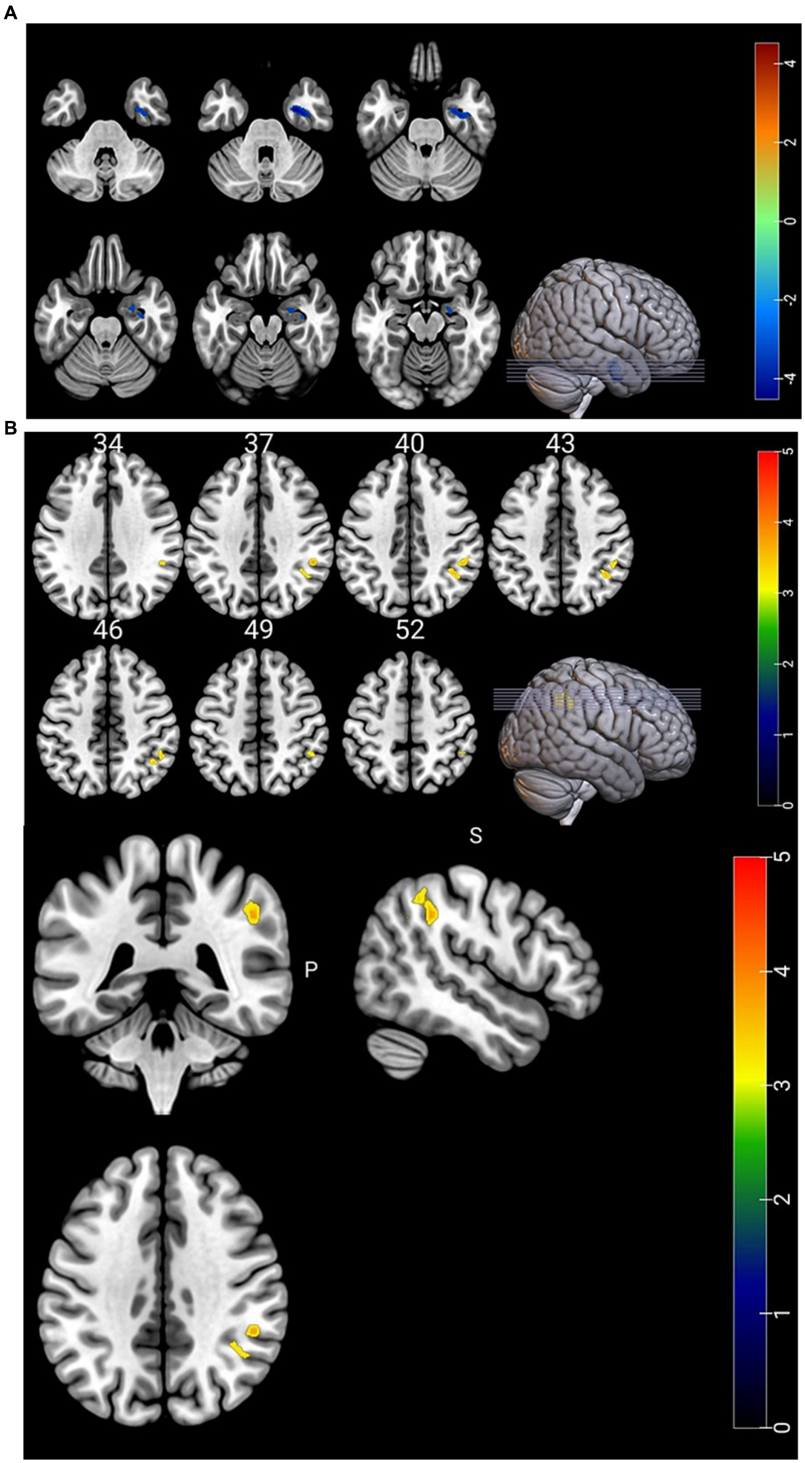
**(A)** Brain regions with differences in CBF. CBF in the right parahippocampal gyrus was significantly lower in the NLE group than in the HC group. **(B)** Brain regions with differences in CBF/ALFF ratio. CBF/ALFF ratio in the right inferior parietal, but supramarginal and angular gyri was significantly increased in the NLE group than in the HC group.

Compared with HC group, CBF in the right parahippocampal gyrus was significantly decreased and CBF/ALFF ratio in the right inferior parietal, but supramarginal and angular gyri was significantly increased in NLE group (*p* < 0.001). There was no significant difference in ALFF between the two groups (*p* > 0.05). The statistically significant results are shown in Table 2 and Figures 1A,B.

**Table 2:** Brain regions with significant group differences in CBF, ALFF and CBF/ALFF ratio.

Brain regions	MNI peak coordinates			*t* value	Cluster size
	*X*	*Y*	*Z*		
CBF NLE < HC ParaHippocampal_R	28	−4	−32	−4.5917	196
CBF/ALFF ratio NLE>HC Parietal_Inf_R	42	−54	45	4.3514	52

MNI: Montreal Neurological Institute; NLE, non-lesional epilepsy; HC, healthy controls; ParaHippocampal_R, right parahippocampal gyrus; Parietal_Inf_R, right inferior parietal, but supramarginal and angular gyri.

### 3.3 Correlation analysis

**Figure 2:**
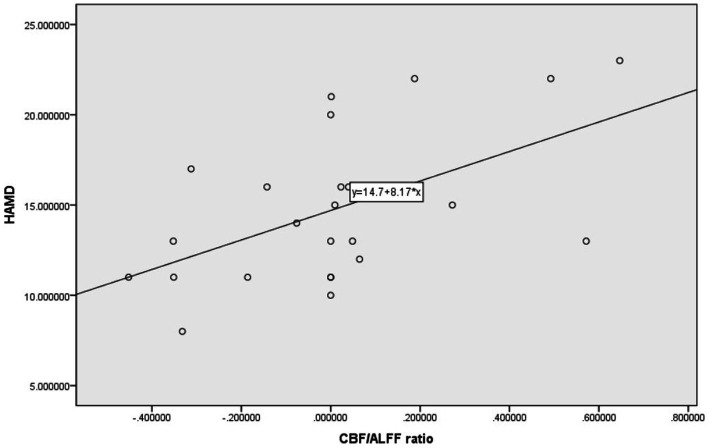
Scatter plot of correlation between HAMD and CBF/ALFF ratio in patients with NLE.

Pearson correlation analysis was used to evaluate the relationship between brain regions with differences in CBF and CBF/ALFF ratio and neuropsychological scores in the NLE group. The results showed that the CBF/ALFF ratio of the right inferior parietal, but supramarginal and angular gyri in the NLE group was positively correlated with the HAMD score (*r* = 0.546, *p* = 0.006), but not significantly correlated with the HAMA score. The statistically significant results are shown in Table 3 and Figure 2.

**Table 3:** Correlation analysis results in patients with NLE (*r* value/*p* value).

	CBF	CBF/ALFF ratio
HAMA	0.072/0.736	0.108/0.617
HAMD	−0.361/0.083	0.546/0.006*

*p < 0.05; CBF, cerebral blood flow; ALFF, amplitude of low-frequency fluctuation; HAMA, Hamilton Anxiety Scale; HAMD, Hamilton Depression Scale.

## 4 Discussion

Previous studies have found that seizures can cause damage to blood–brain barrier (Prager et al., 2019). In this study, we investigated the changes of NVC in patients with NLE by combining BOLD and ASL. The results showed that the CBF in the right parahippocampal gyrus was significantly decreased, and the CBF/ALFF ratio in the right inferior parietal, but supramarginal and angular gyri was significantly increased in NLE patients. Moreover, the CBF/ALFF ratio in the right inferior parietal, but supramarginal and angular gyri was positively correlated with the HAMD score. These findings help us further understand the pathological mechanism of NLE from a new perspective.

The brain is a highly metabolic and energy-consuming organ, accounting for approximately 2% of the total weight of the human body in adults. However, it consumes more than 20% of the body’s total energy, primarily to maintain neuronal activity in the resting state (Lourenço et al., 2018). Previous studies have found that there is a close relationship between neuronal activity and CBF. Increased or decreased CBF can lead to abnormal neuronal activity, and increased neuronal activity will also lead to a significant increase in regional CBF. This phenomenon in the brain is known as NVC (Harris et al., 2014). In 2020, an article published in Nature Review Neuroscience pointed out that NVC provides oxygen and glucose needed to initiate and sustain the neuronal activity, which playing a crucial role in maintaining the steady state of cerebral microcirculation and normal physiological function (Kaplan et al., 2020). The NVU serves as the foundation for the close coupling of neuronal activity and CBF, which is the structural and functional unit of the brain, consisting of neurons, astrocytes, pericytes, smooth muscle cells, etc. (Hu et al., 2019; Kaplan et al., 2020). All the components of the unit are intricately interconnected and mutually interact to maintain the efficient regulation and dynamic equilibrium between neuronal activity and CBF, thereby ensuring the normal physiological function of the brain. In pathological conditions, when the brain neuronal activity and CBF do not match, known as neurovascular decoupling, it can lead to chronic brain injury. The NVC coefficient may either increase or decrease (Zhang et al., 2011; van den Heuvel et al., 2013). Although abnormal changes in neuronal activity and CBF can be found in patients with epilepsy, their spatial distribution does not always align. Therefore, using a single indicator for the evaluation of patients with epilepsy is insufficient. The NVC integrates cerebral blood flow perfusion and neuronal activity related indicators as a whole, and the research in central nervous system diseases is also receiving more and more attention and recognition. Recently, some scholars utilized CBF and ReHo as an overall coupling indicator to investigate the MRI negative epilepsy, revealing significant neurovascular decoupling in patients with this condition (Xu et al., 2023). In our study, ALFF was employed as an indicator to assess the changes of the brain function in epilepsy patients, and the differences in CBF, ALFF and CBF/ALFF ratio between NLE patients and healthy controls were compared. The results showed a significant decrease in CBF in the right parahippocampal gyrus of NLE patients, while the CBF/ALFF ratio of the right inferior parietal, but supramarginal and angular gyri was significantly increased, and the CBF/ALFF ratio of the right inferior parietal, but supramarginal and angular gyri was positively correlated with the epilepsy depression score.

ASL is a water-based magnetic resonance perfusion imaging, which has the advantages of non-invasive, non-radiation, and cost-effective. It is an important method for clinical study of cerebral blood perfusion. Some scholars have found that epilepsy patients have abnormal cerebral blood perfusion, which increases during the epileptic seizures and decreases during the interictal period of epileptic seizures (Matsuura et al., 2015). Martin K et al. (Kojan et al., 2021) discovered that ASL exhibits high sensitivity in preoperative evaluation of epileptogenic foci in MRI negative epilepsy patients. All the studies mentioned above collectively emphasize the significance of ASL in epilepsy evaluation. In our study, compared to healthy controls, NLE patients exhibited significantly lower CBF in the right parahippocampal gyrus, however, the CBF/ALFF ratio did not show significant reduction. The right parahippocampal gyrus is an important component of the limbic system, which is related to emotion and cognition functions. The decrease of CBF in the parahippocampal gyrus suggests that abnormalities within this region may be one of the causes of NLE. Previous studies on medial temporal lobe epilepsy have also demonstrated the significant perfusion reduction within the parahippocampal gyrus among affected patients. According to literature analysis, this phenomenon may be related to the decay of synaptic activity and the decrease of neuron cells in the epileptogenic area (Theodore, 2017).

The inferior parietal, but supramarginal and angular gyri is part of the inferior parietal lobe, which is related to visual and auditory information inputting and processing, language understanding, and episodic memory (Becev et al., 2021; Gray et al., 2020). When damage occurs to this specific brain region, it can lead to abnormal somatosensory circuit. In our study, there were no significant abnormalities in CBF and ALFF in the inferior parietal, but supramarginal and angular gyri in NLE patients, while CBF/ALFF ratio was significantly increased. The CBF/ALFF ratio reflects the change of CBF caused by neuron activity. Under pathological conditions, the NVC function is be reconfigured, which is manifested as the increase or decrease of the coupling coefficient (van den Heuvel et al., 2013). When the CBF and ALFF increase or decrease simultaneously, the CBF/ALFF ratio may not be significantly changed. While CBF increases and ALFF decreases or vice versa, even slight changes can lead to significant differences in the CBF/ALFF ratio, which explains the significant increase of CBF/ALFF ratio in NLE patients despite no significant differences in CBF and ALFF in the inferior parietal, but supramarginal and angular gyri. In addition, our study also found that the alteration in the CBF/ALFF ratio of the inferior parietal, but supramarginal and angular gyri in NLE patients was moderately positively correlated with the epilepsy depression score. The following reasons may exist in our analysis. First, in our study, the NLE patients were younger, and had short onset and lower seizure frequency, so the changes in brain function may not be readily apparent, and there was no significant difference in ALFF between the two groups. Second, the inferior parietal, but supramarginal and angular gyri is an important brain region of the default mode network (DMN) (Long et al., 2021), which plays a crucial role in maintaining physiological activities of the human brain during the rest (Ren et al., 2019). DMN is highly sensitive to energy requirements and blood oxygen changes, making it more susceptible to damage. Previous studies have found that chronic stress events can lead to decreased cortical thickness of brain regions such as the posterior central gyrus and the inferior parietal, but supramarginal and angular gyri (Bartlett et al., 2019), as well as reduced glucose metabolism levels in these regions (Molina et al., 2010). Elizabeth et al.’s study on the depression degree of adolescent girls found that chronic stress events can lead to the decrease of cortical thickness in brain regions such as the posterior central gyrus and the inferior parietal lobule, and the cortical thickness is correlated with the severity of depression. This suggests that abnormal functioning of the inferior parietal, but supramarginal and angular gyri could contribute to changes in depressive mood among patients. Simultaneously, as the CBF/ALFF ratio increases, the level of depression in epilepsy patients gradually rises.

There are some limitations in this study. First of all, the number of case and control groups included in this study is small, which may introduce bias into the research results. The sample size will be increased for further analysis. In addition, all epilepsy patients in this study did not undergo surgical treatment, so the specific type of epilepsy could not be defined. Therefore, we still need to conduct subdivision studies on the type of epilepsy in future work. As an emerging technology in recent years, artificial intelligence is also anticipated to further explore the brain functional network of NLE in future studies (Mofatteh, 2021).

## 5 Conclusion

In conclusion, our study revealed that the CBF in the right parahippocampal gyrus decreased in NLE patients, while the CBF/ALFF ratio significantly increased in the right inferior parietal, but supramarginal and angular gyri, which might be implicated in the neuropathological alterations of NLE. We also found that the CBF/ALFF ratio in the right inferior parietal, but supramarginal and angular gyri may reflect the severity of depression in patients with NLE. Examining brain function changes and cerebral blood perfusion in NLE patients based on ALFF and ASL provides valuable insights into the pathological mechanism of NLE occurrence and development from a new perspective, offering reliable imaging information for clinical diagnosis and treatment.

## Data Availability

The raw data supporting the conclusions of this article will be made available by the authors, without undue reservation.
